# Concise Review: Innate and Adaptive Immune Recognition of Allogeneic and Xenogeneic Cell Transplants in the Central Nervous System

**DOI:** 10.1002/sctm.16-0434

**Published:** 2017-02-28

**Authors:** Chloé J. Hoornaert, Debbie Le Blon, Alessandra Quarta, Jasmijn Daans, Herman Goossens, Zwi Berneman, Peter Ponsaerts

**Affiliations:** ^1^ Laboratory of Experimental Hematology, Faculty of Medicine and Health Sciences, University of AntwerpAntwerpBelgium; ^2^ Vaccine and Infectious Disease Institute (Vaxinfectio), Faculty of Medicine and Health Sciences, University of AntwerpAntwerpBelgium

**Keywords:** Mesenchymal stem cells, Neural stem cells, Transplantation, Immune recognition, Allogeneic, Xenogeneic

## Abstract

Over the last 30 years, numerous allogeneic and xenogeneic cell grafts have been transplanted into the central nervous system (CNS) of mice and men in an attempt to cure neurological diseases. In the early studies, human or porcine embryonic neural cells were grafted in the striatum of animals or patients in an attempt to replace lost neurons. Although the immune‐privileged status of the brain as a recipient organ was widely accepted, it rapidly became evident that CNS‐grafted allogeneic and xenogeneic cells could be recognized and rejected by the immune system, resulting in poor neural graft survival and limited functional recovery. Since then, the CNS transplantation field has witnessed a sharp rise in the number of studies in which allogeneic and xenogeneic neural or mesenchymal stem cells (NSCs or MSCs, respectively) are transplanted, predominantly aiming at providing trophic stimulation and promoting endogenous repair of the brain. Interestingly, in many recent NSC and MSC‐based publications functional improvement was used as the principal measure to evaluate the success of cell transplantation, while the fate of transplanted cells remained largely unreported. In this review, we first attempt to understand why primary neural cell isolates were largely substituted for NSCs and MSCs in cell grafting studies. Next, we review the current knowledge on the immune mechanisms involved in the recognition and rejection of allogeneic and xenogeneic cellular grafts in the CNS. Finally, we propose strategies to reduce graft immunogenicity and to improve graft survival in order to design improved cell‐based CNS therapies. Stem Cells Translational Medicine *2017;6:1434–1441*

**Significance Statement**

Recognition and understanding of the innate and adaptive immune mechanisms involved in immunological rejection of allogeneic/xenogeneic cellular grafts in the central nervous system is a major prerequisite for the design of improved off‐the‐shelf cellular therapies for brain disorders and traumata.

## From Neural Xenotransplantation to Allotransplantation of Neural and Mesenchymal Stem Cells in the Central Nervous System

Before the turn of the century, embryonic neural cells and/or dissociated neural tissue were the main sources of donor material used in central nervous system (CNS) transplantation studies, which predominantly focused on Parkinson's disease and Huntington's disease 1, 2, 3. The ethical concerns associated with the use of human embryos and their limited availability instigated the search for alternative, xenogeneic cell sources. Fetal porcine neural cells were found highly suitable for human transplantation for various reasons. In particular, pigs have large litters, their brains are of a similar size to the human brain and porcine cells are easily amenable to genetic modification 4. Despite some initial successes, it however rapidly became evident that immune‐mediated rejection of xenografts would represent the biggest—if not unsurmountable—hurdle toward achieving successful CNS transplantation, and thus, neural cell replacement. Since then, several promising open‐label clinical trials using allogeneic neural cells were performed, although clinical benefit failed to be reproduced in ensuing double‐blinded trials 5, 6. From 1998 to 2000, Osiris Therapeutics presented a series of studies suggesting that mesenchymal stem cells (MSCs), hematopoiesis‐supporting stromal cells of the bone marrow, could act as immune regulators 7. Specifically, they found that human MSCs suppressed the proliferation of activated T cells and mixed lymphocyte reactions in a major histocompatibility complex (MHC)‐unrestricted, allogeneic manner. This finding was considered a major breakthrough for the field of cell transplantation, seeing that a universal allogeneic MSC preparation could potentially be used to treat a multitude of (chronic) inflammatory conditions in patients. Preclinical evidence additionally revealed a trophic role for MSCs, including—but not limited to—the stimulation of angiogenesis, neurogenesis, and synaptogenesis, as well as the reduction of apoptosis 8. Of note, nearly all these features have also been described for neural stem cells (NSCs), making them equally interesting candidates for neuroprotection and neuroregeneration research 9, 10. The immunomodulatory and trophic stem cell properties of NSCs and MSCs, rather than the cells' multilineage differentiation capacity, greatly encouraged the use of these stem cells for the treatment of a wide array of neuroinflammatory conditions at both the preclinical and clinical levels 11. In the context of this review manuscript, it is important to note that immunomodulatory properties of stem cells on pathology‐associated immune responses, especially in case of allogeneic cell preparations, does not necessarily implicate that grafted stem cells will not be recognized by the host's immune system. Moreover, especially for allogeneic MSC administration we previously demonstrated that different immunological processes are responsible for the recognition and rejection when administered via different routes 12. This review will exclusively focus on the immune mechanisms in play following direct intracerebral or intraspinal administration of allogeneic and xenogeneic cells. In many of the recently conducted preclinical intracerebral cell transplantation studies, functional improvement was used as the principal measure to evaluate the success of cell transplantation, whereas survival rate and immunogenicity of transplanted cells remained largely unreported 13, 14. This observation is rather surprising seeing the prior knowledge on immune recognition of (primary neural) CNS cell grafts. Furthermore, although such cellular therapies have been deemed safe in patients, large placebo‐controlled studies unfortunately have failed to demonstrate therapeutic efficacy 7, 11. It is thus plausible that the challenge to demonstrate efficacy in patients is (partly) attributable to an incomplete understanding of the fate of cellular grafts following transplantation. Accordingly, better insights into this matter would greatly facilitate the search for strategies to prolong cell graft persistence—and thus, its therapeutic window—in vivo.

## Immune Recognition of Cellular Grafts in the CNS

Direct cellular grafting into the CNS is a process that never goes unnoticed by the host's immune system, regardless of the origin of the transplanted cells 15, 16. The phylogenetic distance between cell donor and recipient does however determine whether or not a graft is rejected and how vigorous this process will be 17, 18. In this context, four categories of cell grafts can be identified based on their degree of donor‐recipient mismatch: discordant xenografts (between distantly related species; for example, grafting of human/porcine cells into rodents or grafting of porcine cells into humans), concordant xenografts (between closely related species; for example, grafting of rat cells into mice, or vice versa), allografts (between genetically nonidentical individuals of the same species), and autografts (donor = recipient). This latter category can further be extended to syngeneic grafting, that is, the transplantation between genetically identical individuals of the same species (e.g., inbred rodent strains or identical twins). Immunity against discordant xenografts typically involves components of the innate immune system such as natural antibodies, complement, natural killer (NK) and NKT cells as well as T cell responses. The immune recognition of concordant xenografts and allografts, however, is dominated by T lymphocytes and macrophages/microglia 19, 20. Immunological events associated with autograft (or syngeneic graft) transplantation, that is, the induction of local inflammation and recruitment of phagocytes to the graft site, were found to rapidly subside and may even promote long‐term cell graft survival in the CNS 16, 17, 21, 22, 23.

### The Brain Is Not Immunologically Privileged

The brain has long been considered an immunologically privileged site for transplantation owing to its distinctive architecture: while the (presumed) absence of conventional lymphatics was thought to prevent antigen drainage from CNS tissue to secondary lymphoid organs, the presence of a blood‐brain barrier (BBB) was believed to block immune cell entry into the CNS. Nevertheless, functional lymphatic vessels connecting the meninges to deep cervical lymph nodes were recently discovered, and activated T cells—but not naive T cells or antibodies—can readily cross the BBB in search of their cognate antigen 24, 25. Evidently, both afferent and efferent arms of the immune response to brain antigens are intact. It can however be appreciated that immune recognition of cellular grafts in the CNS is differentially regulated than the recognition of cellular grafts in peripheral non‐neural tissue. In healthy brain, transforming growth factor (TGF)‐β plays a critical role in limiting T cell trafficking in the CNS. This constitutively produced cytokine downregulates the expression of adhesion molecules on endothelial cells of the BBB, thus minimizing leukocyte infiltration 26. Furthermore, TGF‐β suppresses the proliferation of T lymphocytes that do enter the CNS, downregulates MHC class II expression on parenchymal cells and limits the production of monocyte chemoattractant protein (MCP)‐1, a chemoattractant for monocytes/activated T cells and an activator of monocytes, by astrocytes 27, 28, 29. Upon CNS infection or injury, bacterial lipopolysaccharide and/or proinflammatory cytokines such as tumor necrosis factor (TNF)‐α, interferon (IFN)‐γ, and interleukin (IL)‐1β are capable to oppose the action of TGF‐β and enhance immune surveillance of the brain by dramatically increasing the BBB permeability 26. TNF‐α, a cytokine not present in the brain under steady‐state conditions, additionally promotes CNS‐derived antigen drainage and significantly enhances MCP‐1 production by astrocytes 29, 30. Moreover, both TNF‐α and IFN‐γ were found to upregulate MHC expression on astrocytes and oligodendrocytes 31, 32. Inflammation‐driven activation of the BBB endothelium thus augments lymphocyte passage, whereas activation of brain parenchymal cells decreases the threshold required for subsequent immune recognition 33. Together, these data demonstrate that immunological surveillance of the CNS is exquisitely regulated and highly malleable depending on the brain's current inflammatory status.

### Early Inflammatory Responses Following Cell Grafting in the CNS

Having been our laboratory's research focus for many years, we recently proposed a model of the sequential cellular events taking place shortly after intracerebral transplantation of syngeneic mesenchymal cell grafts, reviewed in detail in Le Blon et al. 16. Since (MHC‐unrestricted) innate immune cells were found to dominate the early stages of cell graft recognition, this model can be extrapolated to allografts and concordant xenografts. Early discordant xenograft recognition, however, is expected to be more complex as rapidly acting innate components are protagonists in their immune recognition/rejection process. Owing to the absence of existing blood vessels in cellular grafts, the core of a cell graft becomes severely hypoxic as early as a few hours after transplantation 22, 34. Twenty‐four hours post‐transplantation, hypoxia, and nutrient deprivation—but also anoikis—will have already caused massive cell death in the core of the cell graft, whereas cells near the graft‐brain interface remain viable 22. Hypoxia‐ and anoikis‐induced cell death, rather than subsequent immune responses, is the main cause of the low survival rate (i.e., typically < 30% of grafted cells) associated with cell transplantation in the brain, both for MSC 22, 35, 36, 37 and NSC grafts 38, 39. The danger signals released upon apoptosis/necrosis of the hypoxic cells in turn trigger an early influx of neutrophils within the first 24h after transplantation 22. By day 3 post grafting, phagocytic neutrophils have cleared most of the cell debris in the graft core and the first macrophages/microglia have been recruited to the graft site. This time‐point is also characterized by the onset of neo‐angiogenesis and astroglial scarring in and around the graft, respectively 22, 35. During the following 3‐4 days, a growing number of endothelial cells, microglia and macrophages accumulate in/around the graft and reactive astrocytes increasingly encapsulate the mesenchymal cell graft 22, 36. By day 10, all graft‐infiltrating neutrophils have undergone apoptosis and a dense astrocytic scar separates the (stabilized) cell graft from the surrounding brain tissue 16. Although initially proposed for fibroblast‐like mesenchymal cell grafts (embryonic fibroblasts, MSCs), this model of early cellular events following transplantation also applies to other cell types, notwithstanding some minor cell‐specific differences 35, 38, 40.

### Microglia/Macrophage Response

Along with neutrophils, CNS‐resident microglia and blood‐borne macrophages are the first cells to be recruited to sites of CNS transplantation 17, 40, 41, 42. Correspondingly, MCP‐1 chemokine expression is rapidly upregulated in the brain following cell grafting or vehicle injection 43. Although microglia and macrophages display many functional similarities, both cell types are differentially distributed throughout fibroblast‐like mesenchymal cell grafts: microglia are predominantly found in the graft border, whereas blood‐derived macrophages mainly infiltrate the graft site 16, 21, 44, 45. To our knowledge has not yet been investigated in detail following grafting of NSC. Nevertheless, this may suggest that both cell types exert distinct functions in the immune recognition and/or remodeling of cellular grafts, although further investigation is needed to identify their respective functions 16. In response to pro‐inflammatory cytokines, microglia/macrophages undergo various functional changes: (a) enhanced phagocytic activity, (b) production of nitric oxide and/or superoxide, (c) enhanced surface expression of MHC and costimulatory molecules, and (d) production of regulatory molecules such as cytokines, chemokines, complement components and growth factors 46, 47, 48. Given their capacity for free radical production, phagocytosis and pro‐inflammatory cytokine secretion (i.e., IL‐1β, TNF‐α, and IL‐6), microglia/macrophages can induce direct cell death of transplanted cells, while binding of complement and/or antibodies to foreign material enables these phagocytes to selectively target allogeneic and xenogeneic cells 18, 40, 48. Moreover, microglia/macrophages acquire superior antigen‐presenting capacities upon activation, illustrated by the upregulation of MHC and costimulatory molecules such as CD80 and CD86 [46]. It is however still unclear if antigen‐loaded microglia/macrophages are capable to migrate out of the CNS to initiate T‐cell responses in secondary lymphoid organs, or whether these cells' antigen‐presenting function is mainly restricted to the reactivation of primed T cells at the graft site 49. In case of the latter, alternative ways must exist through which graft‐associated antigens are drained to cervical lymph nodes, such as antigen uptake by dendritic cells 50, 51. In a recent study by our laboratory, we demonstrated that the early recognition/rejection of allogeneic MSCs in the CNS was mediated by microglia/macrophages—but not T cells—whereas MSC allografts in non‐neural tissue induced the activation of alloreactive T cells 12. In addition to their immune‐initiating role, the presence of microglia/macrophages at sites of CNS inflammation may also promote cell graft survival through the production of trophic factors. In this regard, host‐derived microglia/macrophages were found in close association with ingrowing blood vessels and vascular sprouts, suggesting an active role for innate immune cells during graft neovascularisation 23. Finally, microglia/macrophages are key players in the prevention of excessive neuroinflammation through the Fas/Fas ligand (FasL) regulatory axis 46, 52. In healthy CNS, FasL‐expressing microglia induce apoptosis of infiltrating Fas‐expressing T lymphocytes, keeping the CNS trafficking of T cells consistently low. In inflamed CNS, microglia upregulate their surface expression of Fas, thereby increasing their own vulnerability to Fas/FasL mediated apoptosis 46. In conclusion, microglia and macrophages are protagonists in both immune recognition as well as in the remodeling and survival of CNS cell grafts. However, although it may be believed that microglia follow a similar pattern of pro‐inflammatory (M1) and anti‐inflammatory (M2) polarization as compared to macrophages, our most recent data in the context of MSC grafting in the CNS clearly demonstrate that this is an oversimplified statement. Experiments involving intracerebral implantation of IL13‐producing MSC, in order to mediate M2 polarization of MSC graft associated microglia and macrophages (which could be distinguished in either bone marrow chimeric mice or in the CX_3_CR1‐eGFP x CCR2‐RFP transgenic mouse model), clearly demonstrated that the expression of typical M2 markers, like Arginase1, Ym1 and Fizz1, was specifically induced in MSC graft‐infiltrating macrophages, but not (or to a lesser extent) in MSC‐graft recognizing microglia 44, 45. This striking discrepancy in microglia versus macrophage behavior following in vivo stimulation with IL13 was also apparent under pathological conditions, despite responses being more complex dependent on the pathology 44, 45 and/or the methodology 53 applied to deliver IL13 to the CNS.

### T Lymphocyte Response

Considerable experimental evidence points to the involvement of T lymphocytes in the rejection of allogeneic/xenogeneic neural—but not mesenchymal—cell grafts in the CNS: (a) porcine embryonic brain cells induce an in vitro proliferative response in human T cells 54, (b) graft‐infiltrating CD4^+^ and CD8^+^ T lymphocytes are found in rejecting and recently rejected grafts whereas long‐term surviving grafts are (nearly) devoid of T cells 41, 55, (c) improved xenograft survival in animals treated with T cell depleting antibodies or with immunosuppressive drugs known to suppress T cell function 56, 57, 58, (d) indefinite xenograft survival in nude athymic rodents 59, and (e) the blockade of T cell costimulatory pathways prevents discordant xenograft rejection in mice 60. Following their activation in secondary lymphoid organs, T lymphocytes cross the BBB and graft‐specific T cells accumulate at the graft site, whereas nonspecific T cells were found to exit the brain within 48 hours of CNS entry 25. Under normal conditions, the immune response to neural grafts resembles a delayed‐type hypersensitivity reaction of which Th1 CD4^+^ T lymphocytes are the principal mediators. This is reflected by the preferential recruitment of CD4^+^ T cells to the graft site and a coinciding accumulation of Th1 cytokine‐encoding transcripts (i.e., IL‐2 and IFN‐γ) in the CNS 18, 43, 61, 62. Furthermore, neural xenograft survival could be dramatically prolonged following CD4^+^—but not CD8^+^—T lymphocyte depletion 56, 63. In immunoglobulin‐deficient mice, an increased contribution of cytotoxic CD8^+^ T cells to xenograft rejection was observed 62. It can thus be speculated that CD8^+^ T cell‐mediated rejection mainly serves as a redundancy mechanism, which becomes increasingly important when preceding immunological events fail to mediate graft rejection. The prolonged survival of neural xenografts in CD1d1 knockout mice additionally demonstrates a role for NKT cells in CNS graft recognition/rejection 64. In striking contrast with their dominant role in neural graft recognition, the contribution of T cells to the immunological rejection of allogeneic and xenogeneic mesenchymal cell grafts in the CNS seems negligible. This is evidenced by the absence of reactive T lymphocytes in the periphery as well as limited mesenchymal cell graft infiltration by T cells 12, 42. Moreover, treatment of MSC allograft recipients with cyclosporin A, a commonly used T‐cell immunosuppressant, did not prevent rapid cell graft rejection 65. Jointly, these studies demonstrate that microglia/macrophages, rather than T cells, are the principal mediators of MSC immune recognition/rejection in the CNS, while the rejection mechanism of neural allografts and xenografts seems to be highly T cell dependent. Although our own data support this hypothesis, that is, the absence of T cell responses following MSC transplantation in mouse brain, xenogeneic (human) MSC grafted in rat spinal cord were unable to survive without the T cell immune suppressor Cyclosporin A 66. Therefore, also species‐specific and/or graft site‐specific differences in immune responses should be considered in future analyses.

### Antibody‐Mediated Response

While intracerebral cell graft recognition seems to be mediated by microglia, macrophages and T cells, host humoral responses should not be overlooked. Although the intact BBB is impermeable to antibodies, the transplantation procedure temporarily compromises its integrity (i.e., about 7‐12 days for cellular grafts) 67, 68. Correspondingly, antibodies were found to play an initiating role in porcine neurograft rejection 62. Discordant xenografts express highly immunogenic epitopes, for example, the α‐1,3‐galactosyl transferase enzyme on porcine cells, which are recognized by preformed IgM and IgG antibodies. Following cellular transplantation, xenogeneic epitopes rapidly become a target for these natural antibodies that are already present in the serum without need for prior exposure 62, 69, 70. Microglia/macrophage‐mediated phagocytosis (opsonization), NK‐ and microglia/macrophage‐mediated antibody‐dependent cellular cytotoxicity, and antibody‐dependent complement activation are three putative mechanisms through which antibodies are believed to contribute to xenogeneic graft rejection 62, 70, 71, 72, 73, 74. Upon CNS grafting, allogeneic cell grafts induce humoral responses and complement activation 67, 72, 75. In contrast to natural antibodies, no circulating levels of alloreactive antibodies are present at the time of transplantation 67, 75. Discordant xenografts too elicit graft‐specific antibody responses following CNS transplantation, characterized by a delayed appearance of xenoreactive IgG1 and IgG2a antibodies and an accumulation of Th2 cytokine‐encoding transcripts in the brain 18, 61, 73. Even so, the functional role of these allospecific and xenospecific antibodies remains elusive, as BBB integrity is restored before their appearance and as the presence of antibodies does not seem to correlate with graft rejection 67, 68, 75.

### Graft Recognition in Humans

Despite the multitude of immune recognition mechanisms in play, interference with a single pathway (e.g., CD4^+^ lymphocyte depletion or immunoglobulin deficiency) is often sufficient to achieve excellent cell graft survival in rodents 57, 62. In contrast, protocols encompassing multiple immunosuppressive drugs are inadequate to prolong cell graft survival to a similar extent in humans. Contrary to animal studies, which routinely use young nulliparous recipients, patients may previously have been presensitized to allogeneic MHC molecules through prior transplantation, blood transfusion or pregnancy. Despite the exceptional human leukocyte antigen polymorphism, the high frequency of a few alleles significantly increases the odds of presensitized patients harboring memory cells specific for random cell donors 48. At the time of the first clinical studies, fetal neural allografts were considered minimally immunogenic in humans, circumventing the need for (long‐term) immunosuppression 76. More than 15 years later, Krystkowiak and colleagues documented the first case of allograft rejection in a patient transplanted with fetal neural cells 75. In the same study, several other patients had developed signs of donor alloimmunisation without displaying overt signs of clinical or radiological rejection. In contrast, Li et al. found no evidence of an ongoing immune/inflammatory response in a patient grafted with allogeneic embryonic neurons despite prior termination of immunosuppression 77. This high interpatient variability could result from varying degrees of donor‐recipient MHC mismatch among the patients, a factor found to strongly correlate with the magnitude of the elicited alloresponse in rhesus macaques 78. Clearly, additional studies are required to validate this hypothesis and establish why cellular grafts cause alloimmunisation—with or without subsequent rejection—in some patients but not in others. Aside from graft rejection, tumor formation is another important complication of (stem) cell grafting, particularly in immunosuppressed subjects 79. Although both neural and mesenchymal cell grafts have already shown promising results in small patient cohorts, larger‐scale placebo‐controlled studies are warranted to identify the key challenges associated with human CNS cell grafting.

## Strategies Promoting Cell Graft Survival in the CNS

In order to maximize the therapeutic capacity of cellular grafts, several different strategies can be applied to overcome (or minimize) immunological rejection of the grafted cells. Pharmacological suppression of the host immune system is the most commonly used method, with a multitude of immunosuppressants already having been approved for human use 80. Similarly, antibody‐mediated depletion of specific immune components, such as T cells or complement factors, may also prolong graft persistence 80. Both these strategies rely on the modulation of host immunity, leaving the host vulnerable to infections. Alternatively, cell graft persistence can be enhanced by modification of the cell graft itself. To this end, cells are genetically engineered to produce survival‐promoting factors, such as immunomodulatory cytokines or proteins capable of inducing immune cell apoptosis 12, 81, 82. In this context, we have recently shown that interleukin‐13‐expressing MSC allografts survived longer than unmodified MSC allografts in both muscle and brain tissue 12. Instead of relying on the modification of the host or the cells to promote in vivo graft survival, transplanted cells can also be shielded from host immune cells through the presence of a physical barrier 83. Encapsulation of a cellular transplant isolates the graft from the host using a selectively permeable barrier, which allows the bidirectional diffusion of small molecules (e.g., ions, oxygen, carbon dioxide, growth factors, cellular waste products and therapeutic molecules secreted by the grafted cells) while preventing the traffic of cells and large molecules (e.g., immune cells, antibodies and complement). Since the presence of a cell‐impermeable barrier also precludes neo‐angiogenesis in the graft, cografting (or coencapsulation) of oxygen‐releasing biomaterials may be recommended 84.

## The Future of Allotransplantation and Xenotransplantation in the CNS

**Figure 1:**
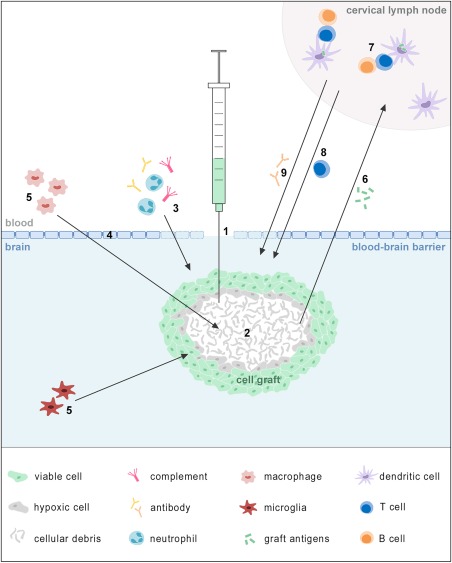
Immune recognition of allogeneic and xenogeneic cell grafts following cell grafting in rodent brain. As a result of the transplantation procedure, blood‐brain barrier (BBB) integrity is immediately compromised **(1)**. In response to the ensuing tissue insult and to hypoxia and anoikis‐induced apoptosis or necrosis of the cell graft core **(2)**, neutrophils, complement elements and natural antibodies are rapidly recruited to the graft site between 6 and 24 hours post grafting **(3)**. Local BBB permeability may further be enhanced for several days (exact timing unknown) as a result of the increasingly pro‐inflammatory environment **(4)**. From on day 3 post grafting blood‐borne macrophages and brain‐resident microglia accumulate, respectively, in and around the graft site **(5)**. Between day 3 to day 14 post grafting (or until removal of all antigens), cellular debris is processed by brain‐resident antigen‐presenting cells that migrate toward the lymph nodes and/or drain to the cervical lymph nodes **(6)** where they are processed by host dendritic cells. From on several days post grafting, in the lymph node, naive allograft/xenograft‐specific T and B cells are activated and proliferate **(7)**. From on 1–2 weeks post grafting, graft‐specific effector T cells **(8)** and alloreactive/xenoreactive antibodies **(9)** may accumulate at the graft site.

Thirty years following the first allotransplantation and xenotransplantation studies in rodents and humans, it is clear that many open questions remain regarding the immunological processes involved in cell graft recognition and, more importantly, the clinical benefit of (allogeneic and xenogeneic) cellular therapies. The lack of scientific support for the latter may not be so unexpected as cell integration/replacement strategies for CNS therapy have been approached with too simplified—even naive—thoughts which are not supported by current knowledge in developmental biology and regeneration. Therefore, future clinical attempts should take into account the multitude of immunological mechanisms that may interfere with graft survival and differentiation (Fig. 1). Furthermore, novel strategies will need to achieve nutrient support and avoid anoikis in order to create a permissive environment for cell graft survival and, ultimately, integration. Clearly, NSC and MSC therapy are applied with very different end goals: whereas functional cell integration is envisioned following NSC transplantation, MSC therapy aims at achieving sustained local cell survival in order to allow long‐term therapeutic factor production. Alternatively, inflammation‐induced repair mechanisms—rather than cell survival itself—may be responsible for (part of) the beneficial effects seen following cellular grafting in the CNS 16, 22. This hypothesis is supported by the demonstration that cell grafting can exert long‐term therapeutic benefit even though just a fraction of grafted cells persists in time 85. Future preclinical and clinical studies will therefore have to reveal which of these hypotheses is backed by scientific and clinical proof. Once this has been established, we believe that the field of allogeneic cell grafting in the CNS will revive and ultimately lead to clinical cell therapy successes for CNS disorders.

## Author Contributions

C.J.H.: conception and design, literature interpretation and discussion, manuscript writing; D.L.B., A.Q., J.D., H.G., and Z.B.: literature interpretation and discussion; P.P.: conception and design, literature interpretation and discussion, final approval of manuscript.

## Disclosure of Potential Conflicts of Interest

The authors indicated no potential conflicts of interest.
